# Hsp90 Stabilizes SIRT1 Orthologs in Mammalian Cells and *C. elegans*

**DOI:** 10.3390/ijms19113661

**Published:** 2018-11-20

**Authors:** Minh Tu Nguyen, Milán Somogyvári, Csaba Sőti

**Affiliations:** Department of Medical Chemistry, Semmelweis University, H-1094 Budapest, Hungary; minhtu.nguyen@med.semmelweis-univ.hu (M.T.N.); somogyvari.milan@med.semmelweis-univ.hu (M.S.)

**Keywords:** aging, *daf-21*, HDAC, stress, proteostasis, signaling, metabolism, epigenetics, *sir-2.1*

## Abstract

Sirtuin 1 (SIRT1) othologs are ubiquitous NAD^+^-dependent deacetylases that act as nutrient sensors and modulate metabolism and stress responses in diverse organisms. Both mammalian SIRT1 and *Caenorhabditis elegans* SIR-2.1 have been implicated in dietary restriction, longevity, and healthspan. Hsp90 is an evolutionarily conserved molecular chaperone that stabilizes a plethora of signaling ’client’ proteins and regulates fundamental biological processes. Here we report that Hsp90 is required for conformational stabilization of SIRT1 and SIR-2.1. We find that inhibition of Hsp90 by geldanamycin (GA) induces the depletion of mammalian SIRT1 protein in a concentration and time dependent manner in COS-7 and HepG2 cells. In contrast to SIRT1, SIRT2 level remains unchanged by GA treatment, reflecting a specific Hsp90 SIRT1 interaction. Hsp90 inhibition leads to the destabilization and proteasomal degradation of SIRT1. Moreover, we observe a GA-sensitive physical interaction between SIRT1 and Hsp90 by immunoprecipitation. We also demonstrate that *hsp-90* gene silencing also induces SIR-2.1 protein depletion and proteasomal degradation in *C. elegans*. Our findings identify metazoan SIRT1 orthologs as Hsp90 clients and reveal a novel crosstalk between the proteostasis and nutrient signaling networks, which may have implications in various age related diseases.

## 1. Introduction

Sirtuins are a divergent family of class III histone deacetylases present in all kingdoms of life [1]. The SIRT1 subfamily, named after mammalian Sirtuin 1 (SIRT1), includes the the most characterized orthologs, such as *Caenorhabditis elegans* SIR-2.1, *Drosophila* dSir2, and the firstly discovered yeast Silent Information Regulator 2 (Sir2) [2]. Mammalian SIRT1 is one of the seven paralogs (SIRT1-7) [1]. SIRT1 is predominantly localized in the nucleus and it deacetylates and regulates beyond histones several dozen proteins involved in genomic stability and signaling. Its substrates include p53, HSF1, FOXO1, 3a and 4, PPARγ, PGC1α, NF-κB, eNOS, LKB1 kinase, and CLOCK/BMAL [3]. Thus, SIRT1 modulates stress responses and survival, metabolism, epigenetic regulation, DNA repair, immunity, neuronal signalling, neuroprotection, cognitive function, synaptic plasticity, and the circadian rhythm [4,5,6]. 

SIRT1 orthologs use NAD^+^ to deacetylate their substrates, hence it appears that the NAD^+^/NADH^+^ ratio is a direct regulator of their activity [7]. Indeed, such a nutrient sensor function might be involved in their effects on aging and health [8]. After the initial finding on the contribution of Sir2p to yeast longevity [9], lifespan extension caused by genetic activation of metazoan SIRT1 orthologs have been challenged [10], yet, their involvement in aging and in the beneficial effects of dietary restriction is supported by several lines of evidence [3]. In mammals, although whole body overexpression of SIRT1 in mice protects from the metabolic syndrome, it does not affect lifespan, whereas overexpression in brain induces longevity [11,12]. Complete SIRT1 loss of function in mice induces high perinatal mortality with severe cardiac and neurological defects [13], while tissue specific SIRT1 knockouts aggravate diet-induced metabolic syndrome [14]. SIRT1 plays a complex role in cancer, depending on the context [15], and is primarily protective in various age-related diseases, such as the metabolic syndrome, neurodegeneration, and inflammation [16]. Therefore, it is not surprising that its function is regulated both at the transcriptional and post-translational level including phosphorylation, sumoylation, methylation, and by protein–protein interactions [14,17]. Allosteric regulation by small molecular sirtuin activating compounds (STACs) make it a druggable therapeutic target with promising clinical trials in progress [6]. The aforementioned effectors not only control SIRT1 activity, but also its stability [18,19]. Intriguingly, both the lack of phosphorylation as well as various stresses, such as covalent modification by oxidants or heat shock similarly induce SIRT1 destabilization, aggregation and proteasomal degradation [20,21,22]. Whether the delicate vulnerability of SIRT1 structure is counterbalanced by a specific conformational maintenance is currently unknown. 

Heat shock protein 90 (Hsp90) is an essential, conserved eukaryotic molecular chaperone [23,24]. Substrates that require Hsp90 for their conformational stability are called “clients” and are highly enriched in multidomain signaling proteins that are intrinsically unstable [25]. To date, several hundred clients are identified and the list is still growing (the list of clients can be found here: www.picard.ch/downloads/Hsp90interactors.pdf). Clients include around 60% of the human kinome including Raf-1, Akt, and LKB1, 7% of transcription factors such as p53, steroid receptors, HSF1, PPARγ, as well as several enzymes and complexes (e.g., calcineurin, eNOS, telomerase, Argonaute, BMAL1). Hsp90, similarly to SIRT1 integrates various signaling pathways [26]. Moreover, yeast Hsp90 is one of the most connected proteins interacting with ~20% of the proteome [25], revealing it as a hub and stabilizer of the protein–protein interaction and signaling network. Accordingly, Hsp90 plays a critical role in a plethora of biological processes that range from proteostasis, stress responses, cell proliferation, motility, immunity, neuronal signaling, circadian rhythm, and further it appears to be involved in epigenetic regulation and the stress-induced canalization of phenotypic variation at the cellular and at the organismal population level [25,27,28,29,30]. Much less is known about the interactions of *C. elegans* Hsp90 ortholog encoded by the *hsp-90* gene (previously called *daf-21*) despite its important roles in development, motility, and longevity [31,32,33,34,35]. 

Hsp90 performs its specific and diverse chaperone function with the dynamic assistance of various of co-chaperone sets and ubiquitin ligases [30,36]. Its chaperone cycle is regulated by ATP binding and hydrolysis [30,36,37,38]. Disruption of the cycle by the specific ATP antagonist geldanamycin (GA) leads to the destabilization and ubiquitinylation of clients, followed by a proteasomal degradation [39,40,41]. Cellular networks exhibit a more stringent dependence on Hsp90 during growth and proteotoxic stress conditions, such as development, fever, cancer, as well as viral and unicellular parasitic infections. Therefore, GA derivatives and other Hsp90 inhibitors offer a therapeutic approach in cancer and in various infectious diseases [42,43,44]. The apparent similarity between the substrates and the physiological processes regulated by SIRT1 and Hsp90, respectively, as well as the indication of structural instability in SIRT1 prompted us to investigate their interaction.

## 2. Results

### 2.1. The Hsp90 Inhibitor Geldanamycin Depletes SIRT1 Protein in Mammalian Cells

**Figure 1:**
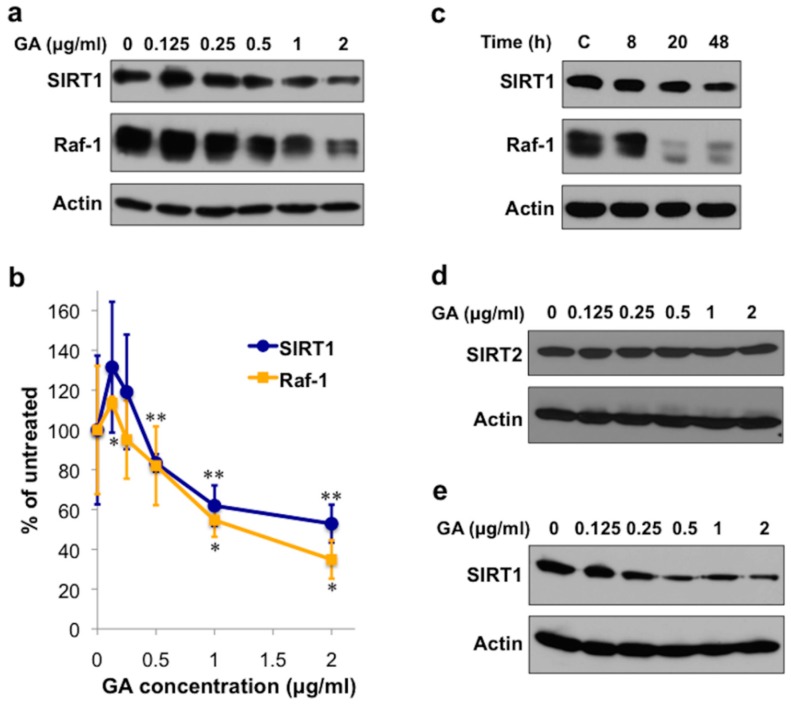
Hsp90 inhibition depletes SIRT1 protein in COS-7 and HepG2 cells. (**a**) Effect of GA on SIRT1 and Raf-1 protein levels. Western blots of lysates from COS-7 cells treated by GA for 20 h. Images are representatives of three experiments for SIRT1 and two experiments for Raf-1. (**b**) Quantification of protein levels from the experiment shown in panel (**a**). Values are means ± S.D. of three (SIRT1) or two (Raf-1) experiments and were statistically compared with the respective untreated controls. * *p* < 0.05, ** *p* < 0.01 by two-tailed unpaired *t*-test. (**c**) Timescale of GA treatment on SIRT1 and Raf-1 protein levels. Western blots of lysates from COS-7 cells treated by 1 μg/mL GA for the indicated times. Images are representatives of two experiments. C: 48 h vehicle control. (**d**) Effect of GA on SIRT2 protein level. Western blots of lysates from COS-7 cells treated with GA for 20 h. Images are representatives of two experiments. (**e**) Effect of GA on SIRT1 protein level in human hepatoma cells. Western blots of lysates from HepG2 cells treated by GA for 48 h. Images are representatives of two experiments.

First, we investigated the stability of mammalian SIRT1 in response to Hsp90 inhibition. We found that a 20-h treatment of the COS-7 monkey kidney cell line by increasing concentrations of GA after an initial increase caused a ~50% decrease in SIRT1 protein (Figure 1a,b). It also decreased the level of Raf-1, a well-known Hsp90 client [40] with comparable concentration dependence (Figure 1a,b). Timescale experiments indicated a decay of SIRT1 protein, although somewhat slower compared to Raf-1, characteristic to Hsp90 clients (Figure 1c). Similarly to SIRT1, SIRT2 also exhibits a cytosolic/nuclear localization, shares the conserved middle catalytic domain, but lacks the extensive N- and C-terminal domains [45]. Therefore, we tested, if the Hsp90 antagonist induced depletion is specific to SIRT1, or may also involve SIRT2. However, GA did not affect SIRT2 protein levels, showing a selective sensitivity of SIRT1 protein to the disruption of the ATP dependent chaperone function of Hsp90 (Figure 1d). Next, we asked if SIRT1 level was also sensitive to GA in HepG2 human hepatoma cells and it was (Figure 1e). Thus, Hsp90 specifically stabilizes SIRT1 protein in two different mammalian cell lines.

### 2.2. Disruption of the Hsp90-SIRT1 Interaction Leads to Destabilization and Proteasomal Degradation of SIRT1

**Figure 2:**
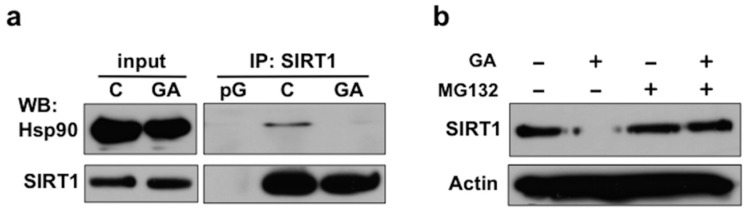
Disruption of the Hsp90-SIRT1 interaction leads to destabilization and proteasomal degradation of SIRT1. (**a**) SIRT1 physically interacts with Hsp90 in a GA-sensitive manner. Western blots showing the co-precipitation of Hsp90 with SIRT1 from COS-7 cells treated by 1 μg/mL GA or vehicle for 3 h. pG, protein G control; C, control (DMSO vehicle). (**b**) GA induces destabilization and proteasomal degradation of SIRT1. Western blots of total cell lysates from cells treated by 1 μg/mL GA and/or 5 μM MG132 or DMSO vehicle for 20 h. Images are representatives of two experiments.

Next, we investigated whether a physical interaction exists between SIRT1 and Hsp90. To this end, we immunoprecipitated endogenous SIRT1 protein from COS-7 cells. Hsp90 was co-precipitated with SIRT1, and their complex was disrupted by a 3-h GA treatment (Figure 2a). To test, if in the absence of Hsp90, SIRT1 is degraded by the proteasome, we simultaneously treated cells by GA and/or the proteasome inhibitor MG132 and prepared total cell lysates using urea and SDS to solubilize the detergent insoluble fraction. While MG132 alone did not influence SIRT1 level, in response to a combinatorial inhibition of both Hsp90 and the proteasome, SIRT1 re-appeared in the lysate indicating that upon disruption of its interaction with Hsp90, SIRT1 is destabilized and degraded by the proteasome (Figure 2b). Altogether, these results demonstrate that Hsp90 associates with SIRT1 and ensures its conformational stability, identifying SIRT1 as an Hsp90 client protein.

### 2.3. Hsp90 Is Required for SIR-2.1 Protein Stability in Caenorhabditis elegans

**Figure 3:**
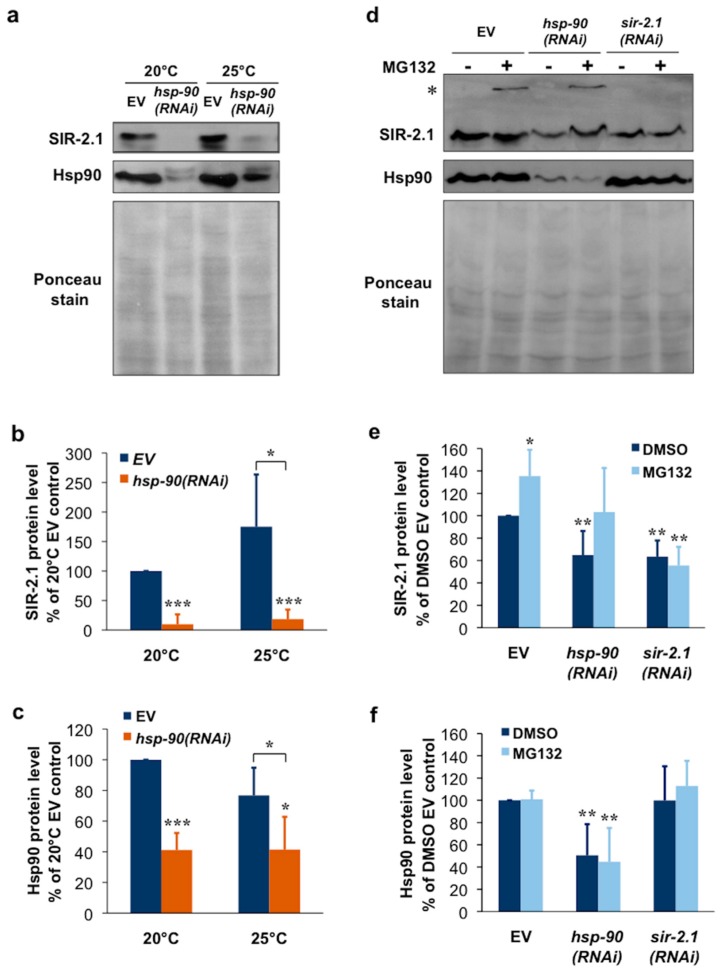
Hsp90 knockdown induces SIR-2.1 protein depletion and proteasomal degradation in *C. elegans*. (**a**) Effect of *hsp-90(RNAi)* on SIR-2.1 and Hsp90 protein levels. Western blots of lysates from young adult N2 wildtype nematodes treated with *hsp-90(RNAi)* or empty vector (EV) from hatching. Worms were kept at 20 or 25 °C. Images are representatives of three experiments. (**b**,**c**) Quantification of SIR-2.1 and Hsp90 protein levels from the experiment shown in panel (**a**). Values are means ± S.D. of three experiments and were statistically compared with the respective EV controls. * *p* < 0.05, *** *p* < 0.001 by two-tailed unpaired *t*-test. (**d**) *hsp-90(RNAi)* induces destabilization and proteasomal degradation of the SIR-2.1 protein. Western blots of total lysates from young adult N2 wildtype nematodes treated with *hsp-90(RNAi)*, *sir-2.1(RNAi)* or empty vector (EV) from hatching and transferred to plates at the L2 stage containing 10 µM MG132 or DMSO vehicle. Images are representatives of four experiments. Asterisk (*) indicate anti-SIR-2.1 reactive protein aggregates accumulated on the top of the gel in the MG132-treated EV and *hsp-90(RNAi)*, but not the *sir-2.1(RNAi)* lanes. (**e**,**f**) Quantification of SIR.2.1 and Hsp90 protein levels from the experiment shown in panel (**d**). Values are means ± S.D. of four experiments and were statistically compared with the respective controls. * *p* < 0.05, ** *p* < 0.01 by two-tailed unpaired *t*-test.

We asked whether the chaperone-client interaction between Hsp90 and SIRT1 could be extended to the nematode *C. elegans*. To this end, we silenced Hsp90 by feeding worms with an *hsp-90(RNAi)* [35,46] (Figure S1a,b). A 48-h *hsp-90(RNAi)* treatment induced a ~60% reduction in Hsp90 protein and caused an almost quantitative, ~90% depletion of SIR-2.1 protein at the ambient temperature, 20 °C (Figure 3a–c). At 25 °C SIR-2.1 showed a 50%, non-significantly increased protein level and a similar magnitude of reduction in response to Hsp90 knockdown (Figure 3a–c). *hsp-90(RNAi)* treatment of a low copy *sir-2.1* transgenic strain and its background strain [47] outcrossed in our lab [10] both yielded comparable results (Figure S1c,d). To address if SIR-2.1 protein is degraded by the proteasome we treated nematodes with MG132 and prepared total worm lysates using urea and SDS to solubilize the detergent insoluble fraction. In these experiments *hsp-90(RNAi)* depleted SIR-2.1 protein by 40%, similarly to *sir-2.1(RNAi)*, perhaps due to the presence of DMSO. MG132 treatment increased SIR-2.1 protein level in EV fed worms. Furthermore, MG132 treatment restored SIR-2.1 level to EV control level in combination with *hsp-90(RNAi)*, but not with *sir-2.1(RNAi)* which downregulates *sir-2.*1 mRNA (Figure 3d–f). Moreover, in some of the experiments a small portion of SIR-2.1 protein was unable to enter and accumulated on top of the resolving gel, only in response to proteasome inhibition of EV and *hsp-90(RNAi)*, but not *sir-2.1(RNAi)* treated samples. These observations suggest a proteasome-mediated turnover and destabilization of SIR-2.1 in physiological conditions, which is further aggravated by a reduction in Hsp90 availability. We hypothesize that Hsp90 capacity in non-stress conditions might already limit SIR-2.1 folding and function. Altogether our findings indicate that Hsp90 is required for the structural stabilization of SIR-2.1 protein in *C. elegans*.

## 3. Discussion

Our study identified the evolutionarily conserved Hsp90 chaperone as a conformational stabilizer of mammalian and nematode SIRT1 orthologs. The dependence of clients on Hsp90 is determined not by specific motifs, but by their thermodynamic instability. Therefore the Hsp90 clientele exhibits a continuum from weak to strong interactions over a 100-fold affinity range [26]. The GA sensitivity of mammalian SIRT1 approaches that of Raf-1, a very strong Hsp90 client [26] (Figure 1a,b) indicating a stringent dependence of SIRT1 on Hsp90 in COS-7 cells. Likewise, *hsp-90* silencing entirely depleted SIR-2.1 in nematodes.

**Figure 4:**
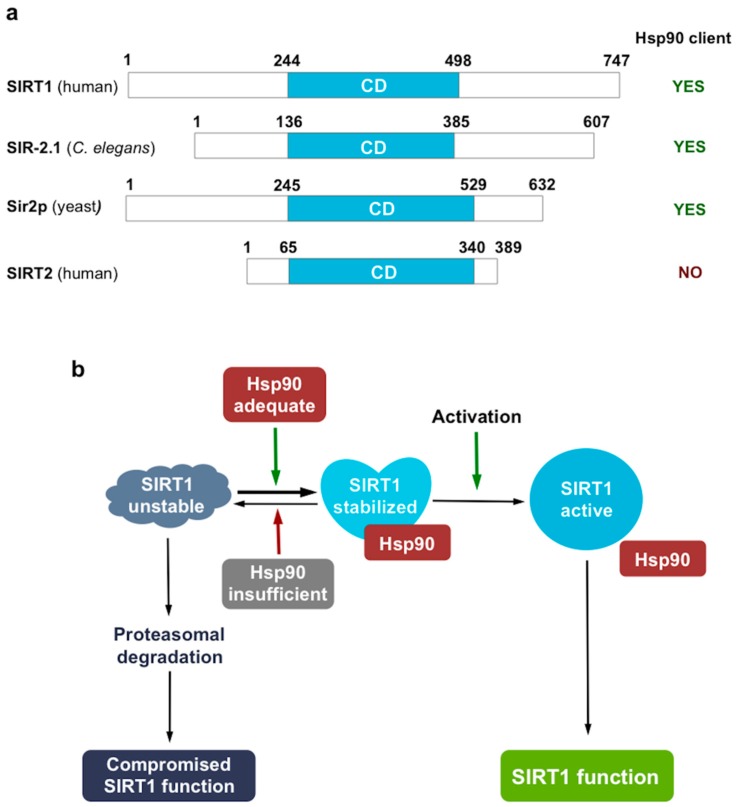
A conserved chaperone-client interaction between Hsp90 and SIRT1 orthologs. (**a**) SIRT1 orthologs possess extensive N- and C-terminal domains. Domain structures of yeast, worm, and human SIRT1 orthologs and the SIRT1 paralog SIRT2. Numbers denote the number of amino acid residues at the respective domain boundaries. The catalytic core domains (CD) are in light blue, N- and C-terminal regions are in white. (**b**) Hypothetical model of the conformational regulation of SIRT1 (and respective orthologs) by Hsp90. Hsp90 binds the nascent, unstable SIRT1 in the cytosol and stabilizes it in a probably open, partially unfolded conformation. Hsp90 binding facilitates various interactions which ensure activation by signals, substrate, and co-factor binding. Active, closed SIRT1 might dissociate from, or might remain in loose complex with, Hsp90 and exerts its cellular functions. Reduction in Hsp90 capacity, by pharmacological inhibition, genetic down-regulation or stresses, induces SIRT1 destabilization and proteasomal degradation, which in turn attenuates SIRT1 mediated processes.

We found that in contrast to SIRT1 and SIR-2.1, Hsp90 does not influence SIRT2 protein stability (Figure 1c). Hsp90 clients in general are larger, more difficult to express in a heterologous expression system in soluble form and more difficult to obtain their crystal structures, than nonclients [26]. Out of the seven mammalian and four *C. elegans* sirtuin paralogs, SIRT1 and SIR-2.1, respectively, stand out by possessing two-fold longer protein sequences (747 aa. for SIRT1 vs. 314–400 aa. for SIRT2-7; (607 aa. for SIR-2.1 vs. 287–294 aa. for SIR-2.2-2.4) and harboring extensive N- and C-terminal regions besides the conserved 250 aa. catalytic middle domain [48]. They share these similarities with all SIRT1 orthogs including the yeast Sir2p (662 aa. [2] Figure 4a). Intriguingly, an earlier study showed that Hsp90 is required for Sir2p protein levels and *sir2*-mediated gene silencing in yeast [49]. Hitherto crystal structures for all full length sirtuins except SIRT1 and SIRT7 have been solved [50,51,52,53,54]. However, attempts to solve the structure of the full length SIRT1 have not been successful. Only the catalytic domain [55] as well as an engineered mini-SIRT1 protein harboring the 183–229 N-terminal and the 641–665 C-terminal segments [56] yielded high resolution crystallographic structures. In agreement with the experimental data, the catalytic core domain performs an essential function and is structurally highly conserved from yeast to mammals, while the N- and C-terminal segments are variable both in length, sequence and secondary structure [48]. These observations indicate that the catalytic core adopts a stable optimal fold, while the other regions are optimized to play a specific and regulatory role in the individual sirtuin enzymes function and have a higher degree of freedom across evolution. Such a property is characteristic to Hsp90 clients which allows them a greater conformational space for flexibility, a key to induced, regulated interactions. Besides, the extensive, evolutionarily non-restricted sequences allow the emergence of new interactions at the expense of protein instability, which is chaperoned by Hsp90 [26]. Based on the findings on the selective stabilization of Sir2p [49], SIR-2.1 and SIRT1, but not SIRT2 (this study), by Hsp90, we propose that the requirement of SIRT1 orthologs for Hsp90 mediated stabilization involve region(s) beyond the catalytic core (Figure 4a). These findings also suggest that the ancestor of SIRT1 sirtuins became dependent on Hsp90 at dawn of eukaryotic life.

Interestingly, phosphorylation affects SIRT1 stability and proteasomal degradation. Phosphorylation by JNK2 at Ser27 stabilizes [18], whereas persistent JNK1 activity at Ser47 destabilizes [19]. Ser47 phosphorylation has also been reported upon oxidative stress induced SIRT1 protein depletion [20]. On the other hand, in the absence of DYRK1A and DYRK3 mediated Thr530 phosphorylation at the C-terminal domain, SIRT1 aggregates both in vitro and in vivo [22], providing an independent confirmation for the involvement of both the N- and the C-terminal regions in SIRT1 stability. The interaction of the various phosphorylated forms of SIRT1 with Hsp90 is a relevant question for further studies. 

The major function of Hsp90 is to hold its client in an activation competent state, be it a steroid receptor until ligand binding, a kinase until ATP and substrate arrives, telomerase or Argonaute until the RNA is loaded, which was hypothesized to stabilize clefts and surfaces for interaction to occur [26,57]. Indeed, recently obtained cryoelectron microscopic structure of the CDK4 kinase bound to Hsp90 [58] shows extensive interactions along multiple regions, stabilizing a client in an unexpectedly open, unfolded state. Such an open state is a more favorable way to transit between inactive and active conformation(s) and also allows various signals and posttranslational modifications to happen in a more fluent manner than a closed conformation [58]. The apo form of the SIRT1 catalytic domain is also in an open conformation exposing the hydrophobic interior which closes upon substrate and cofactor binding [59]. Likewise, the SIRT1 catalytic domain is subject to various regulatory interactions originating from the N-terminal as well as the C-terminal domains, which are modulated by diverse signals, including allosteric sirtuin activator binding, phosphorylation, and other modifications [6,17,48,56]. Hence, Hsp90 might co-ordinate several steps in the SIRT1 activation process, such as ensuring proper folding, binding of NAD^+^ and substrate and finely tuning it with activatory/inhibitory signals and interaction partners. We propose a hypothetical model integrating our experiments with literature data (Figure 4b). Whether this model holds true and Hsp90 binding involves extensive interactions with several SIRT1 regions and keeps SIRT1 in an open conformation, remains to be determined.

Our findings using the specific Hsp90 inhibitor GA demonstrate that a reduction in functional Hsp90 availability compromises SIRT1 stability. Therefore, when using Hsp90 inhibitors in tumor chemotherapy one might need to consider the potential impact of SIRT1 inhibition especially in tumors where SIRT1 suppresses malignancy [15,44]. Due to the significant overlap between SIRT1 substrates [4,6] and Hsp90 clients [25,30], the physiological outcome of the alteration of SIRT1 function will require further carefully designed studies. Nevertheless, the Hsp90 SIRT1 complex may be an important hub in the integration of stress responses.

We have previously shown that proteotoxic stresses, such as heat shock and proteasome inhibition diminish the availability of Hsp90 and reversibly halt adipocyte differentiation via destabilizing the Hsp90 clients PPARγ and Akt [29]. Such a stress-responsive, immediate regulation of cellular function and phenotype by client stability might also involve the similarly stress-induced SIRT1 orthologs. Our hypothesis is supported by a study showing the destabilization and proteasomal degradation of SIRT1 during heat shock, which contributes to p53 hyperacetylation and apoptosis [21]. SIRT1 proteins are regulated by the nutrient supply through the level of NAD^+^ [6], whereas Hsp90 is responsive to the cellular ATP/ADP ratio [25,38]. The Hsp90 dependent regulation offers an opportunity for SIRT1 function to integrate inputs both from proteostasis as well as from the ATP level. Likewise, efficiency of SIRT1 activators might require abundant Hsp90 and co-chaperone capacity, which decreases during aging but can be upregulated by chaperone inducers [60]. Such a combination therapy might benefit several age-related metabolic and protein misfolding diseases, where SIRT1 activation holds great promise [6]. Interestingly, we have recently reported that Hsp90 is required for *C. elegans* longevity by both DAF-16/FOXO isoform A dependent and independent mechanisms [35]. Hence, it is plausible and remains to be seen whether Hsp90 might modulate lifespan via SIR-2.1/SIRT1.

## 4. Materials and Methods

### 4.1. Materials

Reagents for cell culture were from Gibco-Invitrogen (Carlsbad, CA, USA) and for *C. elegans* maintenance were Sigma (St. Louis, MO, USA). Rabbit polyclonal antibodies against SIRT1 and SIRT2 were from Cell Signaling Technology (Danvers, MA, USA). Mouse mondoclonal anti-Raf-1 was from BD Transduction Laboratories (San Jose, CA, USA), mouse monoclonal anti-β-actin was from Sigma (St. Louis, MO, USA), mouse monoclonal anti-Hsp90 was from Institute of Immunology Ltd. (Tokyo, Japan). Rabbit polyclonal antibodies against *C. elegans* Hsp90 [61] and SIR-2.1 [62] were kindly provided by Eileen Devaney (University of Glasgow, Glasgow, Ireland) and Anton Gartner (University of Dundee, UK), respectively. HRP-conjugated secondary antibodies were from Dako (Agilent Technologies, Santa Clara, CA, USA). Geldanamycin (GA) was from Sigma, the proteasome inhibitor MG132 was from Calbiochem (San Diego, CA, USA). Complete protease inhibitor tablets were from Roche (Mannheim, Germany). Protein assay, electrophoresis and blotting reagents were from Bio-Rad (Hercules, CA, USA). The ECL kit was from Perkin-Elmer (Wellesley, MA, USA). All other reagents were from Sigma or Fluka (Buchs, Switzerland).

### 4.2. Cell Culture

COS-7 (SV40-transformed African green monkey kidney fibroblast-like cell line) and HepG2 human hepatoma cells were obtained from the ATCC (Manassas, VA, USA). Both cell types were maintained in Dulbecco’s modified Eagle medium (with 4.5 mg/mL glucose), supplemented with 10% fetal bovine serum, 2 mM l-glutamine, 1.5 g/L sodium bicarbonate, 4.5 g/L glucose, 100 μg/mL streptomycin and 100 IU/mL penicillin at isobaric oxygen in 5% CO_2_ at 37 °C. 

### 4.3. Mammalian Cell Lysis

Cell lysis and western blotting was essentially as described [29]. Cells were lysed with ice-cold lysis buffer (50 mM Tris, 300 mM NaCl, 0.5 mM EDTA, 0.1 mM EGTA, 1 mM MgCl_2_, 1% NP40, 20% glycerol, 0.5 mM DTT, 2× Complete, pH 7.6) at 4 °C for 20 min. Cells were vortexed vigorously, and centrifuged at 13,000 rpm for 10 min. Protein concentration of the supernatants was determined by the Bradford method. Proteasomal degradation of SIRT1 was assessed by making total cell lysates in urea buffer (2% SDS, 6 M urea, 30 mM Tris, 2× Complete, pH 7.6) using the same procedure as above. Protein concentration was determined by the detergent-compatible Pierce BCA protein assay (Thermo Scientific, Wiesbaden, Germany). 

### 4.4. Western Blotting

Protein extracts were run on SDS-PAGE then transferred to nitrocellulose membrane. Membranes containing *C. elegans* protein samples were stained with Ponceau. Membranes were blocked in 5% (*w*/*v*) skim milk powder. Then, blots with mammalian protein samples were probed with rabbit polyclonal antibodies against SIRT1 (1:1000) and SIRT2 (1:1000), mouse monoclonal antibodies against Raf-1 (1:2000) and β-actin (1:10,000) at 4 °C overnight. Blots with *C. elegans* protein samples were probed with a rabbit polyclonal antibody against Hsp90 (1:1000) or SIR-2.1 (Batch 1434.3, 1:1000) at 4 °C overnight. After washing, blots were incubated with horseradish peroxidase (HRP)-conjugated secondary anti-rabbit or anti-mouse antibodies, respectively (Dako, Agilent Technologies, Santa Clara, CA, USA) for an hour at room temperature and developed by ECL. Mammalian protein levels were determined by the Image J software (NIH, Bethesda, MD, USA) and normalized using the respective β-actin densities. We note that actin was unreliable as a loading control in the *C. elegans* experiments, therefore protein levels were normalized to total protein lane densities stained by Ponceau.

### 4.5. Immunoprecipitation

5 × 10^6^ cells were treated by 1 μg/mL geldanamycin (GA) or DMSO for 3 h. Then, cells were washed three times and scraped in ice cold PBS. Lysis was performed in IP lysis buffer (50 mM Tris, 2 mM EDTA, 100 mM NaCl, 1 mM Na_3_VO_4_, 1% NP40, 2× Complete, pH 7.6). 1200 μg total protein per sample were used for immunoprecipitation. SIRT1 was immunoprecipitated by a monoclonal anti-SIRT1 antibody. Pellets were washed five times with IP lysis buffer and analyzed by SDS-PAGE and immunoblotting with anti-Hsp90 and anti-SIRT1 antibodies.

### 4.6. C. elegans Strains and Maintenance

All strains used were originally obtained from CGC. Animals were kept at 20 °C using standard *C. elegans* techniques [63]. *Caenorhabditis elegans* strains used in this study: N2: wild type, VC199: *sir-2.1(ok434)*, SCS003: *pkIs1642 [sir-2.1 unc-119] unc-119(ed3)* (outcrossed ×6); SCS004: *pkIs1641 [unc-119] unc-119(ed3)* (outcrossed ×6) [10].

### 4.7. RNA Interference

HT115(DE3) *E. coli* strains producing double stranded RNA against *hsp-90* [46] and *sir-2.1*, respectively, were kind gifts from Eileen Devaney (University of Glasgow, UK) and Tibor Vellai (Eötvös Loránd University, Budapest, Hungary). RNAi feeding was performed using the standard method [64]. *E. coli* clones harboring RNAi plasmids were grown overnight at 37 °C in LB medium containing 100 µg/mL ampicillin. Nematodes were grown on plates containing 1 mM IPTG, 50 µg/mL ampicillin and 6.25 µg/mL tetracyclin, seeded with *E. coli* L4440 empty vector (EV) control and specific RNAi vectors, respectively, from hatching. Measurements were made after two days.

### 4.8. C. elegans Lysis

Synchronized populations of worms were grown on 10 cm NGM plates with IPTG seeded with *E. coli* HT115 harboring either *hsp-90(RNAi*), *sir-2.1(RNAi)* or empty vector (EV). Worms were washed using M9 buffer three times and frozen at −80 °C. Samples were thawed on ice in 200 µL of nematode lysis buffer (50 mM Tris-HCl, 0.25% SDS, 1% NP40, 150 mM NaCl, 1 mM EDTA, 2× Complete, pH 7.4). Proteasomal degradation of SIR-2.1 was assessed by making total worm lysates in nematode lysis buffer supplemented with 6 M urea. Samples were sonicated on ice 6 times for 10 s after three freeze–thaw cycles and centrifuged for 10 min at 10,000× *g* at 4 °C. Protein concentration in the supernatants was determined by the detergent-compatible Pierce BCA protein assay (Thermo Scientific, Wiesbaden, Germany).

### 4.9. Statistical Analysis

Data were compared by two-tailed unpaired *t*-test. Variables were expressed as mean ± standard deviation (S.D.). Statistical significance was indicated as follows: * *p* < 0.05, ** *p* < 0.01, *** *p* < 0.001.

## Supplementary Materials

Supplementary materials can be found at http://www.mdpi.com/1422-0067/19/11/3661/s1.

## Abbreviations

Akt	Ak thymoma kinase, protein kinase B
ATCC	American Type Culture Collection
BMAL1	brain and muscle ARNT-like 1
CDK4	cyclin dependent kinase 4
COS-7	CV-1 in origin SV40 transformed cells
DYRK	dual-specificity tyrosine phosphorylation-regulated kinase
ECL	enhanced chemiluminescence
eNOS	endothelial nitric oxide synthase
FOXO	forkhead box O transcription factor
GA	geldanamycin
HepG2	hepatoma G2
HSF1	heat shock transcription factor 1
Hsp90	heat shock protein 90
*hsp-90*	*C. elegans* heat shock protein 90 gene
JNK	Jun N-terminal kinase
LKB1	liver kinase B1
NAD^+^	nicotinamide adenine dinucleotide
NF-κB	nuclear factor κB
PGC1γ	PPARγ co-activator 1α
PPARγ	peroxisome proliferator-activated receptor-γ
Raf-1	rapidly accelerated fibrosarcoma-1 kinase
SIRT1	sirtuin 1, Mammalian silent information regulator ortholog 1
Src	Rous sarcoma virus thyrosine kinase
STAC	small molecular sirtuin activating compound
