# Supplementary material for: Hsp90 Stabilizes SIRT1 Orthologs in Mammalian Cells and C. elegans
Source: Int J Mol Sci. 2018 Nov 20;19(11):3661. doi: 10.3390/ijms19113661 (PMC6274930; doi:10.3390/ijms19113661)
Supplement: Supplementary file 1 [file ijms-19-03661-s001.pdf]

International Journal of *Molecular Sciences*

MDPI

Supplementary Material

Hsp90 stabilizes SIRT1 orthologs in mammalian cells and *C. elegans*

Minh Tu Nguyen, Milán Somogyvári and Csaba Sőti*

Department of Medical Chemistry, Semmelweis University, Budapest, Hungary, H-1094

*Correspondence: soti.csaba@med.semmelweis-univ.hu;
Tel.: + 36 1 4591500 extn. 60130

This file includes: Supplementary Figure S1 and Legend.

Int. J. Mol. Sci. 2018, 19, 3661; doi:10.3390/ijms19113661

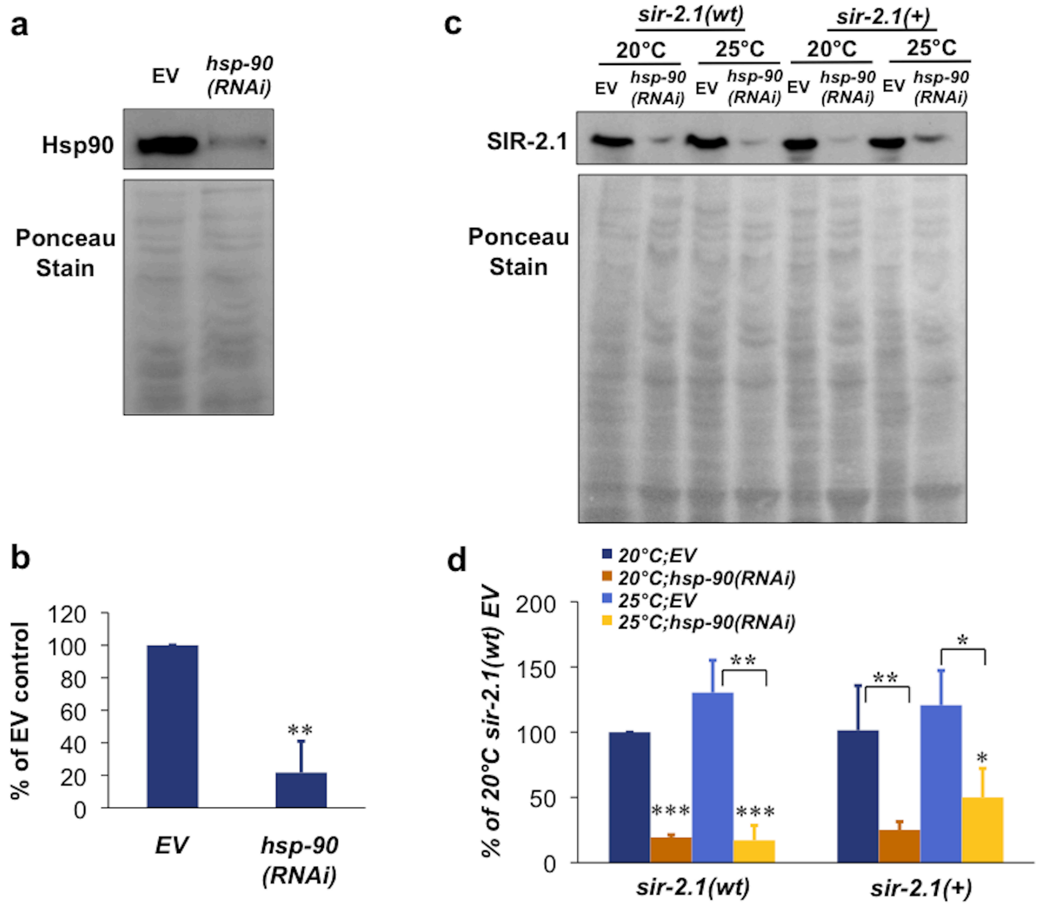

Figure S1 Hsp90 knockdown depletes SIR-2.1 protein levels in a low copy overexpressor *C. elegans* strain. **(a)** *hsp-90(RNAi)* downregulates Hsp90 protein level. Western blots of lysates from young adult N2 wildtype nematodes treated with *hsp-90(RNAi)* or empty vector (EV) from hatching. Worms were kept at 20°C. Images are representatives of three experiments. **(b)** Quantification of protein levels from the experiment shown in panel (a). Values are means ± S.D. of three experiments and were statistically compared with the EV control. *P<0.01, by two-tailed unpaired t-test. **(c)** *hsp-90(RNAi)* depletes SIR-2.1 protein in the low copy transgenic *sir-2.1* and its background strain. Western blots of lysates from young adult SCS004 *sir-2.1(wt)* and SCS003 *sir-2.1(+)* nematodes treated with *hsp-90(RNAi)* or empty vector (EV) from hatching. Worms were kept at 20 or 25°C. Images are representatives of three experiments. **(b)** Quantification of protein levels from the experiment shown in panel (a). Values are means ± S.D. of three experiments and were statistically compared with the respective EV controls. *P<0.05, **P<0.01, ***P<0.001 by two-tailed unpaired t-test.

© 2018 by the authors. Submitted for possible open access publication under the terms and conditions of the Creative Commons Attribution (CC BY) license (http://creativecommons.org/licenses/by/4.0/).
